# Sex‐specific differences in mitochondria biogenesis, morphology, respiratory function, and ROS homeostasis in young mouse heart and brain

**DOI:** 10.14814/phy2.13125

**Published:** 2017-03-22

**Authors:** Abdel Rahman M. Khalifa, Engy A. Abdel‐Rahman, Ali M. Mahmoud, Mohamed H. Ali, Maha Noureldin, Saber H. Saber, Mahmoud Mohsen, Sameh S. Ali

**Affiliations:** ^1^ Center for Aging and Associated DiseasesHelmy Institute of Medical SciencesZewail City of Science and TechnologyGizaEgypt

**Keywords:** Brain, EPR, heart, mitochondria, NADPH Oxidase, ROS, Sex, superoxide dismutase

## Abstract

Sex‐specific differences in mitochondrial function and free radical homeostasis are reported in the context of aging but not well‐established in pathogeneses occurring early in life. Here, we examine if sex disparity in mitochondria function, morphology, and redox status starts early and hence can be implicated in sexual dimorphism in cardiac as well as neurological disorders prevalent at young age. Although mitochondrial activity in the heart did not significantly vary between sexes, female brain exhibited enhanced respiration and higher reserve capacity. This was associated with lower H_2_O_2_ production in female cardiac and brain tissues. Using transmission electron microscopy, we found that the number of female cardiac mitochondria is moderately greater (117 ± 3%, *P* = 0.049, *N* = 4) than male's, which increased significantly for cortical mitochondria (134 ± 4%, *P* = 0.001, *N* = 4). However, male's cardiac mitochondria exhibited fragmented, circular, and smaller mitochondria relative to female's mitochondria, while no morphologic sex‐dependent differences were observed in cortical mitochondria. No sex differences were detected in Nox2 and Nox4 proteins or O_2_‐consuming/H_2_O_2_‐producing activities in brain homogenate or synaptosomes. However, a strong trend of increased EPR‐detected NOX superoxide in male synaptosomes hinted at higher superoxide dismutase activity in female brains, which was confirmed by two independent protocols. We also provide direct evidence that respiring mitochondria generally produce an order‐of‐magnitude lower reactive oxygen species (ROS) proportions than currently estimated. Our results indicate that sex differences in mitochondrial biogenesis, bioenergetics, and morphology may start at young age and that sex‐dependent SOD capacity may be responsible for differences in ROS homeostasis in heart and brain.

## Introduction

Multiple lines of evidence show that mitochondrial dysfunction is associated with increased respiratory impairments and reactive oxygen species (ROS) production that are hallmarks of the etiopathogenesis of several neurological (Panov et al. 2007), as well as cardiovascular maladies (Madamanchi and Runge 2007). For example, enhanced oxidation of cysteine residues within complex I has been reported in a mouse model of Parkinson's disease (PD) (Danielson et al. 2011). Reduction in the expression of mitochondrial electron transport complex genes has been documented in autistic brains (Anitha et al. 2013), and in attention deficit hyperactivity disorder (ADHD) (Marazziti et al. 2012). Attenuation in mitochondrial complex I activity has been reported in ischemic/reperfused rat heart (Solaini and Harris 2005). Mitochondrial dysfunction is also implicated in the pathogenesis of atherosclerotic plaques (Sobenin et al. 2013). In addition to the mitochondria, ROS are also produced by other cellular systems. Of particular importance, the nicotinamide adenine dinucleotide phosphate (NADPH) oxidase (NOX) is increasingly recognized as an important source of oxidative stress in diseases and aging (Altenhofer et al. 2012). The involvement of ROS produced by NOX in triggering neurodegeneration in PD (Taetzsch and Block 2013) and Alzheimer's disease (AD) (Ansari and Scheff 2011) has been documented. Similarly, the role of NOX as a source of oxidative stress in cardiac insults is well characterized (Kleikers et al. 2012).

On the other hand, epidemiological studies have shown that many neurodegenerative etiologies such as PD and amyotrophic lateral sclerosis (ALS) are male predominant (Van Den Eeden et al. 2003; Kim et al. 2012). Recently, a strong male bias in several neurodevelopmental disorders including autism spectrum disorder (ASD) (Werling and Geschwind 2013) and ADHD (Davies 2014) has been also consistently observed. Moreover, prevalence studies demonstrate that cardiovascular diseases such as myocardial infarction and atherosclerosis are steadily underrepresented in premenopausal women compared to their male counterparts (Barrett‐Connor 1997). Although the mechanisms underlying sexual dimorphism in developing these disorders are uncertain, sex differences in biomarkers of oxidative stress have been proposed in clinical and experimental studies. It has been shown that biomarkers of oxidative stress are apparently lower in healthy young women than in age‐matched men (Ide et al. 2002; Powers et al. 2002). Male wistar rats produce more ROS than their age‐matched females (Borras et al. 2003). It is currently known that there is sex‐related difference in multiple key sources of ROS such as mitochondria, cytokines, and NOX enzymes during aging (Dugan et al. 2009; Ali et al. 2010). However, sex differences in mitochondrial function and NOX dynamic in young animals are not fully investigated yet, presumably due to the subtleness of such differences. Moreover, sex differences in oxidative stress and underlying causes in brain and heart are not adequately investigated. Defining relative contributions of different ROS sources (e.g., NOX enzymes vs. mitochondria and Nox2 vs. Nox4) that are responsible for the sex difference in young animals may open new avenues for therapeutic approaches to sexually dimorphic disorders occurring early in life.

Our goal in this study is to analyze subtle, early in life, sex‐dependent differences in mitochondrial function and ROS dynamics in mice brain and heart using an array of sensitive techniques. We compared the activity of different mitochondrial complexes in male and female C57BL6 mice using high‐resolution respirometry. Transmission electron microscopy analyses were employed to probe sex‐specific differences in mitochondrial biogenesis and morphology in cardiac as well as cortical tissues. We also compared NOX activity in male and female synaptosomes by spin‐trapping EPR spectroscopy while monitoring NADPH‐induced oxygen consumption as well as H_2_O_2_ production using the Oroboros O2k Station equipped with a fluorescence detection module. The differential protein expression of Nox2 and Nox4 subunits in female and male brain homogenate and synaptosomes was assessed. We also compared the activity of the antioxidant enzyme superoxide dismutase activity (SOD) in male and female brains utilizing two independent methods including a new EPR methodology. In addition to detecting significant metabolic differences between sexes, our results highlight inherent differences in several key sources of ROS between young males and females.

## Materials and Methods

### Animals

Age‐matched C57BL6 male and female mice (6 weeks old) were purchased from Misr University for Science and Technology (Cairo, Egypt), and were housed for at least 1 month in Zewail City animal facility until they were killed. All animals were maintained in pathogen‐free, ventilated cages in 12‐h light/12‐h dark cycles at 24°C and 50% relative humidity, with free access to water and standard laboratory rodent chow. After exposure to diethyl ether or isoflurane in a closed desiccator inside a well‐ventilated fume hood (open drop method), anesthetized animals were decapitated following quick cervical dislocation. All experiments were conducted in adherence to the guidelines by the U.S. NIH Institutional Animal Care and Use Committee and comply with the animal ethics checklist provided by the Journal of Physiology.

### Mitochondrial respiration

#### Homogenate preparation

After cervical dislocation, whole brain and heart were dissected and immersed in ice‐cold mitochondrial respiration medium, MIR05 containing 110 mmol/L sucrose, 60 mmol/L K‐lactobionate, 0.5 mmol/L EGTA, 1 g/L BSA essentially fatty acid free, 3 mmol/L MgCl_2_, 20 mmol/L taurine, 10 mmol/L KH_2_PO_4_, and 20 mmol/L HEPES adjusted to pH 7.1 at 37°C. Tissues were then transferred to a filter paper to remove excess liquid. A wet weight (WW, 9 mg of forebrain, and 4.5 mg of left atrium) was determined with a microbalance. Tissues were then transferred into Eppendorff tubes containing 2.4 mL of ice‐cold, fresh MIR05. Using a pair of sharp forceps, tissues were cut into small pieces and transferred with the medium into an FT500‐PS Shredder Pulse Tube for use with the PBI‐ shredder (Oroboros Instruments, Innsbruck, Austria). Tissue‐dependent homogenization was then carried out according to the manufacturer's instructions.

#### Measurements of mitochondrial respiratory rates

Mitochondrial respiratory assessments (and hydrogen peroxide determinations) were carried out at 37°C using high‐resolution respirometry system Oxygraph‐2K (Oroboros Instruments, Innsbruck, Austria). Before, starting the experiment, oxygen calibration was performed by allowing the respiration medium MIR05 to equilibrate with air in the oxygraph chambers while stirred at 540–560 rpm for 30–40 min, until a stable signal was detected. 4.5‐mg brain, or 2.2‐mg heart homogenized tissue was then added to be followed by tissue permeabilization with 25‐*μ*g/mL saponin. Substrate–uncoupler–inhibitor titration (SUIT) protocol was performed as follows: Chamber A: Pyruvate (5 mmol/L), Malate (2 mmol/L), and glutamate (10 mmol/L), (PMG) → adenosine diphosphate (ADP, 1.5 mmol/L) → Rotenone (R, 0.5 *μ*mol/L) → Succinate (S, 10 mmol/L) → Antimycin A (AmA, 2.5 *μ*mol/L) → N,N,N',N'‐tetramethyl‐p‐phenylenediamine (0.5 mmol/L)/Ascorbate (2 mmol/L), (TMPD/Asc); and chamber B: PMG + S → carbonyl cyanide m‐chloro‐phenyl hydrazine (CCCP) (several 0.5 *μ*mol/L infusions) → AmA. The rates of oxygen consumptions were calculated as the negative time derivative of oxygen concentration and normalized to tissue wet weight. Electron transport through complex I and III were distinguished by adding rotenone (0.5 *μ*mol/L) and AmA (2.5 *μ*mol/L), respectively. The residual O_2_ flux after inhibition with AmA (O_2_ flux independent of the electron transfer system) was deducted from the values of each of the former steps. The following mitochondrial respiratory parameters were determined (Ali et al. 2010): Leak respiration oxygen flux accomplished after adding PMG with no ADP present (Leak‐I, State 4), (Ali et al. 2006). OXPHOS capacity (state 3), which occurs in the presence of saturating ADP (OXPHOS‐I) or after inhibiting complex I by rotenone and adding succinate as a complex II substrate (OXPHOS‐II), (Altenhofer et al. 2012). Electron Transfer system (ETS) capacity: CCCP titrations was performed in order to determine uncoupled respiration. Respiratory control ratio (RCR) was calculated as the ratio between state 3 (OXPHOS capacity) and the leak state (state 4). Flux control ratios (FCR), were calculated by dividing Leak‐I, Leak‐II, OXPHOS‐I, and OXPHOS‐II values by that of the ETS state. The rate of hydrogen peroxide formation was detected in parallel to oxygen consumption in the same sample. Horseradish peroxidase (HRP, 1 U/mL) and Amplex Red fluorescent dye were utilized. The excitation wavelength was 525 nm and fluorescence detection was at 587 nm. Signals were calibrated using known amounts of hydrogen peroxide that were exogenously added by the end of each run. Data acquisition and analysis were performed with the DatLab^®^ software, version 4.3 (Oroboros Instruments). This enables continuous monitoring and recording of the oxygen concentration in the chambers as well as of the derived oxygen flux over time, normalized for the amount of homogenized tissue acquired at rates of 0.5–1 Hz.

### Transmission electron microscopy

Immediately after killing the animals, small pieces were taken from brain and heart of males and females and fixed in 5% cold glutaraldehyde for 24 h. The specimens were then washed in 3‐4 changes of cacodylate buffer (pH 7.2) for 20 min in each change and post‐fixed in cold osmium tetroxide for 2 h. Then, the specimens were washed in four changes of cacodylate buffer for 20 min each. Dehydration was done using ascending grades of ethyl alcohol (30%–50%–70%) each for 2 h followed by 90% and 100% alcohol for 30 min each. Specimens were then cleared in propylene oxide and embedded in Epon 812 using gelatin capsules. For Polymerization, the embedded samples were kept in the incubator at 35°C for 1 day, then at 45°C for another day and in the 60°C during the third day. Ultrathin section (50–80 nm) from selected areas were made and collected on copper grids. The ultrathin sections were contrasted with uranyl acetate for 10 min and lead citrate for 5 min, and examined by Jeal 100× transmission electron microscope and photographed at 80 kV (Assiut University Microscopy Unit).

### Analyses of mitochondrial morphology

Heart and brain mitochondrial content and morphology were analyzed for four animals per group where 5–7 slides/organ/animal were inspected. Methods for analyzing mitochondria were adapted from (Koopman et al. 2006). Briefly, using ImageJ software (ImageJ, NIH) images were uniformly adjusted for brightness/contrast and mitochondria were manually selected by the freehand tool by two independent blinded operators. A threshold was applied to each image equally and individual particles (mitochondria) were analyzed for circularity, aspect ratio (the ratio between major and minor axes of the analyzed ellipses), area, and perimeter. Form factor (F), a measure of mitochondrial length was determined for each image using the equation F = (perimeter^2^/(4*π* × area)). For mitochondrial morphology, the analysis was performed on 676 male cardiac, 969 female cardiac, 1254 male cortical, and 817 female cortical mitochondria from four animals per group. To study mitochondrial fragmentation, area distributions were analyzed by histogram analysis. Histograms of mitochondrial area versus counts were produced by splitting the area data range into bins of equal size (0.02 *μ*m^2^) for frequency counts. Frequency counts for each bin represent the number of mitochondria from the dataset that fall within the range of each bin. For final graphical representation, frequency counts were converted into percentage mitochondria population calculated per the total number of mitochondria analyzed for each group and plotted against center bin values.

### Isolation of synaptosomes

Isolation of synaptosomes was performed as previously described (Behrens et al. 2007). Briefly, brains were quickly removed, forebrains were dissected, and homogenized using a potter homogenizer in ice‐cold isolation buffer (0.32mol/L sucrose, 1 mmol/L EDTA, 10 mmol/L Tris‐HCl buffer, pH 7.4, 10 mmol/L glucose). To minimize proteolysis, protease inhibitors (0.01 mg/mL leupeptin, 0.005 mg/ml pepstatin A, 0.10 mg/mL aprotinin, 5 mmol/L benzamide) were included in isolation buffer. The homogenate was then centrifuged at 3100 *g* for 3 min at 4°C. The supernatant was removed and the pellet was resuspended in half the volume of isolation buffer, then homogenized again and recentrifuged. The supernatants were collected and mixed with percoll to a final concentration of 15%. The mixture was then layered onto a step gradient of 23% and 40% percoll. Centrifugation was then performed at 30,000 *g* for 5 min at 4°C. The uppermost band was collected, rinsed in isolation buffer, followed by centrifugation and resuspension in synaptosomal buffer (120 mm NaCl, 4.7 mmol/L KCl, 2.2 mm CaCl_2_, 1.2 mm MgCl_2_, 25 mmol/L HEPES, 1.2 mmol/L MgSO_4_, 1.2 mmol/L KH_2_PO_4_, 10 mmol/L glucose).

### Determination of NOX activity in synaptosomes by high‐resolution oxymetry

Nox activity in synaptosomal preparations was determined by measuring NADPH‐induced oxygen consumption and the rate of hydrogen peroxide formation simultaneously using Amplex Red fluorescence in the same sample. Synaptosomal protein was added to the respiration medium in the chamber. The rates of oxygen consumption and hydrogen peroxide formation were measured as described above.

### Determination of NOX activity in synaptosomes by EPR spectroscopy

Measurement of NOX activity was carried out by detecting the NADPH‐dependent superoxide production in synaptosomal fractions using spin‐trapping EPR spectroscopy. A mixture containing 20 *μ*L freshly isolated synaptosomes, 0.22 mol/L DMPO (5,5‐Dimethyl‐1‐pyrroline N‐oxide) spin trap, 5.55 mmol/L DETC to inhibit SOD, and a final concentration of 6.66 mmol/L NADPH was finally added to trigger NOX activity before the sample was loaded in a glass capillary and introduced into the EPR cavity of a MiniScope MS400 Benchtop spectrometer (Magnettech, Berlin). The observed DMPO‐OH signals arising from DMPO‐OOH spin adduct were monitored over 15 min. The area under curve for each kinetic measurement was then calculated over the 15‐min period using OriginPro software and hence taken to represent ROS yields due to NOX oxidative bursts. EPR signals were normalized by protein concentrations of purified synaptosomes as determined by the Bradford assay.

### Western blot

For western blotting, 30 *μ*g‐proteins/lane from forebrain lysates (homogenate supernatant) or isolated synaptosomes (membrane fraction) were resolved by 10% SDS‐PAGE and transferred to PVDF membranes (Millipore). Membranes were blocked with 5% nonfat dry milk in PBS, containing 0.01% Tween 20 for 1 h, and then incubated with anti‐Nox2 (1:200; Santa Cruz Inc.), anti‐Nox4 (1:200; Santa Cruz Inc.), and anti‐ *β*‐actin (1:1000; Santa Cruz Inc.) at room temperature for 2 h. Membranes were then probed with corresponding anti‐rabbit or anti‐goat HRP conjugated secondary antibodies for 1 h (1:2000; Deko, Denmark) and developed using the enhanced chemiluminescence (ECL) reagent (Pierce). Bands were quantified by densitometric analyses using Quantity 1 analysis software (Bio‐Rad, Rockford, USA). The densities of each band, which represented individual animals, were normalized to *β*‐actin and then compared with control levels for both male and female groups.

### Determination of superoxide dismutase activity in brain homogenates

#### Sample Preparation

Mice were decapitated and their brains were rapidly removed and rinsed with cold phosphate buffer saline. Forebrains were dissected and homogenized in ice‐cold buffer (0.32mol/L sucrose, 1 mmol/L EDTA, 10 mmol/L Tris‐HCl buffer, pH 7.4, 10 mmol/L glucose). The homogenized solution was then centrifuged at 1500 *g* for 5 min at 4°C. The supernatant was collected and used for evaluation of the SOD activity.

### Spectrophotometric determination of superoxide dismutase activity in brain homogenate

Superoxide dismutase activity was determined by a method described elsewhere (Kuthan et al. 1982), with slight modifications. Superoxide anion radical (O_2_^•^‾) was generated through aerobic enzymatic metabolism of hypoxanthine or xanthine by xanthine oxidase (X/XO). The [O_2_^•^‾] was spectrophotometrically followed by monitoring the SOD‐inhibitable reduction of ferricytochrome *c* (cyt c) at 550 nm using BMG FLUOstar Omega (BMG Labtech, Germany). The rate of cyt c reduction in the absence and presence of variable protein concentrations of homogenized brain tissue samples from males or females were determined. The determined rates of superoxide generation in the presence of homogenates were then quantified as percentage of that in their absence. We included 120 U catalase in all mixtures to prevent reverse oxidation of ferrocytochrome c by the resulting H_2_O_2_.

### Determination of superoxide dismutase activity in brain homogenate by EPR spectroscopy

To determine the antioxidant capacity of tissue samples against different ROS, we developed a methodology utilizing the ability of EPR spectroscopy to determine different radical species simultaneously. In the present case, we focused on the superoxide dismutase activity of the studied samples but the method is applicable in cases where multiple free radical species are involved. Controlled generation of O_2_^•^‾ by the X/XO system in the presence of the DMPO spin trap allowed the quantification of superoxide yield by EPR in the absence and presence of different protein concentration of each sample (female, *n* = 6; male, *n* = 6). This resulted in dose–response curves, which were analyzed to obtain the IC50 values, that is, protein concentrations required to quench 50% of the controlled superoxide signals by each sample.

### Statistical analysis

Data are presented as means ± SEM. Statistical significance was assessed by applying a one‐way Analysis of Variance (ANOVA) in the OriginPro 8.0. Tukey corrected post hoc tests were carried out in order to identify differences between sexes or organs at all measured parameters. The level of statistical significance was set at *P *<* *0.05.

## Results

### No sex differences in mitochondrial respiratory rates and coupling in the heart

**Figure 1:**
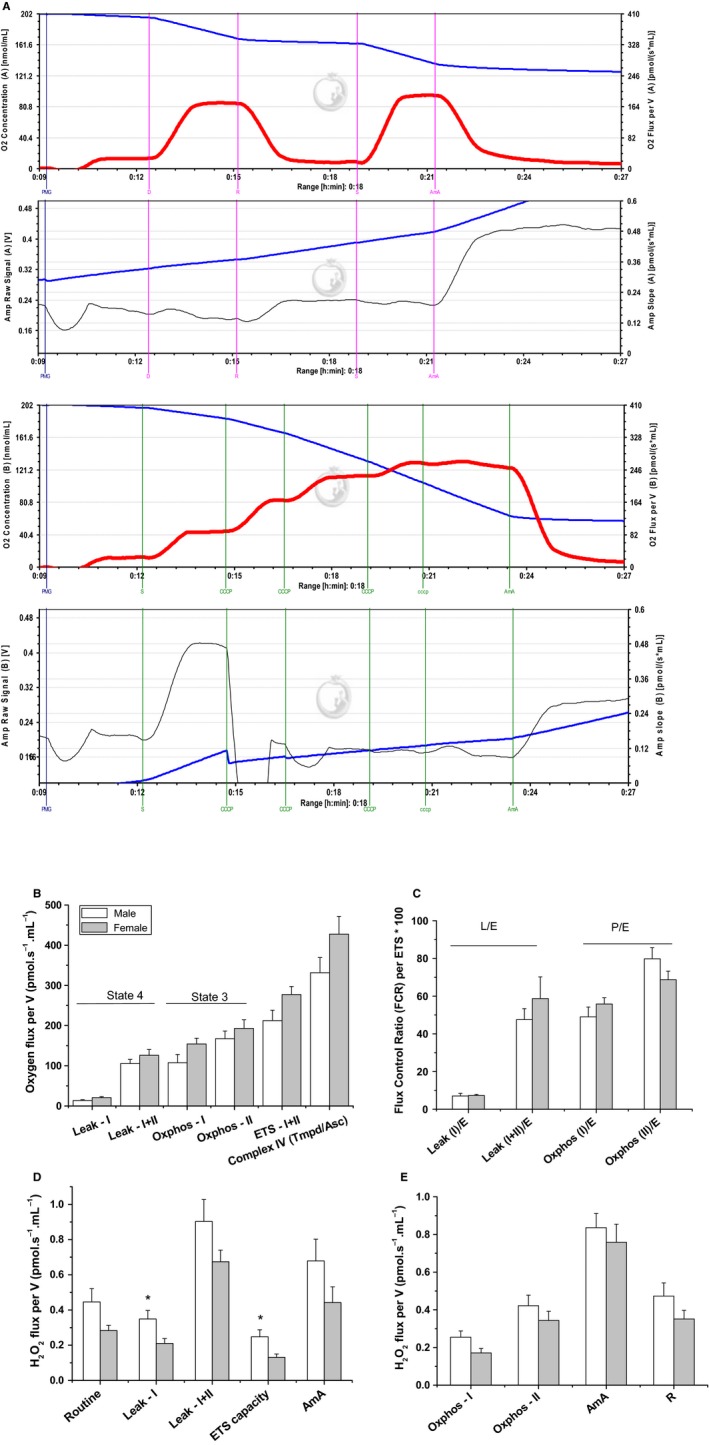
Sex‐dependent high‐resolution mitochondrial oxygen consumption and parallel hydrogen peroxide production in homogenized cardiac tissue. (A) Representative traces depicting ETC substrate‐specific O_2_ utilization (first and third panels from top) and H_2_O_2_ production (second and fourth panels) in homogenized and saponin‐permeabilized female heart during Substrate‐Uncoupler‐Inhibitor Titration (SUIT) as described in the Methods Section. In each panel, both of the level (blue traces) and flux rate (red for oxygen and black for H_2_O_2_). Equal amounts of homogenized tissue were introduced to the two chambers of an Oroboros O2K Oxygraph, allowing the application of two different protocols while simultaneously monitoring resorufin fluorescence resulting from HRP‐mediated H_2_O_2_ oxidation of Amplex^®^ Red in each chamber. The first protocol is performed to interrogate various ETC complexes in terms of their contributions to O_2_ utilization and H_2_O_2_ leakage (upper two panels) and the second is to determine the respiratory reserve capacity and ROS leakage (lower two panels). (B) The rates of oxygen consumption were calculated as the negative time derivative of oxygen concentration (oxygen flux per volume) and normalized to tissue wet weight. Substrates and inhibitors that were combined in each state are listed below the graph. (C) Flux Control Ratio (FCR) per Electron Transfer system (ETS) capacity of complex I and II at Leak and OXPHOS states obtained by dividing oxygen flux at different mitochondrial states by the maximum flux that mitochondria can reach (ETS capacity). Values are expressed as percentages of the ETS‐state. (D) Respiratory Control Ratio (RCR) of complex I obtained by dividing oxygen flux at OXPHOS‐I state by oxygen flux at Leak‐I state. (E&F) Rates of H_2_O_2_ production in pmol/sec*ml during various resting, active, or inhibited respiratory states in the presence of the listed substrates/inhibitors combinations. Values are graphed as mean ± SEM (*n* = 6–8, *P <* *0.05).

Mitochondria are generally assumed to be major sources of ROS production. Because alterations in mitochondrial respiratory function and coupling have been shown to modify ROS production, we evaluated mitochondrial respiration in heart homogenate of 2–4‐month‐old male and female C57BL6 mice. Experiments typically involved parallel measurements of oxygen consumption by identical amounts of a given homogenate sample using two independent SUIT (substrate‐uncoupler‐inhibitor titration) protocols as detailed in the Methods' Section. Representative traces for oxygen consumption and simultaneous H_2_O_2_ production from males cardiac mitochondria are given in Figure 1A. Although females' mitochondrial activity was slightly higher, no significant sex differences in both state 4 (*Leak‐I* or *Leak‐I*+*II*,* n* = 6 per group, Fig. 1B) and state 3 (*OXPHOS‐I or OXPHOS‐II*) were observed. Maximally uncoupled respiratory rate induced by the addition of CCCP, with electron input through both complex I and II was also independent of sex (Fig. 1B). As also shown in Figure 1B, mitochondria from hearts of male and female mice have significantly greater oxygen flux during *Leak‐I*+*II* and *OXPHOS‐II* states as compared to *Leak‐I* and *OXPHOS‐I* states, suggesting more efficient function of complex II in the electron transport chain of cardiac mitochondria.

The FCR is the ratio of oxygen flux during different respiratory states normalized by maximum flux (ETS‐state) and is used to assess the relative contribution of each respiratory state, for example, through complex I and/or II, to the maximal electron transport system capacity. The RCR is the classical parameter for the mitochondrial qualitative control, which is calculated as state 3 divided by state 4 respiration and reflects the OXPHOS coupling efficiency. In our hands, both FCR and RCR were not sex‐dependent. However, Figure 1C also reveals that the maximal electron flux capacities in male and female heart mitochondria involved greater contributions from complex II relative to complex l. In all experiments, RCR was greater than 8, indicating a high degree of integrity of the studied mitochondria. When exogenous cyt c was added on respiring mitochondria, no significant increase in the OCR was observed indicating the integrity of the outer mitochondrial membrane.

### Resting respiratory states leak more ROS in male's heart

To follow the effect of sex on mitochondria‐induced ROS production during physiologically relevant respiratory states, we detected H_2_O_2_ production by O2k‐Fluoremeter utilizing the HRP/Amplex Red fluorescence assay (Fig. 1D,E). H_2_O_2_ is a membrane‐permeable ROS and is mainly generated from catalytic dismutation of superoxide by superoxide dismutase. Figure 1D shows that the rate of H_2_O_2_ production during state 4 (*Leak‐I*) in cardiac mitochondria is higher in males (*n* = 6). These results indicate that no sex difference in ROS leakage during phosphorylating respiration (Fig. 1E) with a male tendency to generate more hydrogen peroxide during resting respiratory states (Fig. 1D). AmA produced the predictable large increase in mitochondrial ROS production, thus confirming that enhanced mitochondrial ROS flux could be identified whenever present (Fig. 1D). Moreover, addition of rotenone during phosphorylating respiration, attenuated AmA‐induced H_2_O_2_ flux (Fig. 1D); presumably by blocking ROS leakage due to reverse electron transfer at complex I (Turrens 2003).

### Enhanced mitochondrial respiratory function in female brain mitochondria

**Figure 2:**
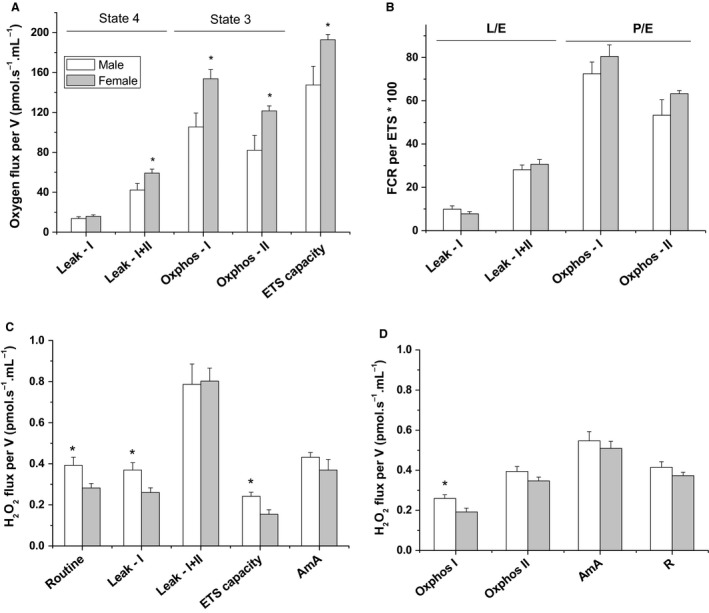
Sex‐dependent high‐resolution mitochondrial oxygen consumption and parallel hydrogen peroxide production in homogenized mouse brain. Analyses of brain mitochondria function and ROS production as described in Fig. 1. Legend. While female brains showed an enhanced mitochondrial respiratory function, male brains exhibited elevated rates of H_2_O_2_. Values are graphed as mean ± SEM (*n* = 6–8, *P <* *0.05).

To test whether sex affects brain mitochondrial function and parallel ROS production, we investigated the same respiratory parameters as described for the heart mitochondria above. Female brain mitochondria exhibited consistently significant increase in state 3 both during *OXPHOS‐I* and *OXPHOS‐II* phosphorylating states (*n* = 9 for males and eight for females; Fig. 2A). Similar trend was observed in female brain mitochondria during *Leak‐I + II* state, but not during *Leak‐I* state suggesting that female brain mitochondria rely more on Complex II (succinate dehydrogenase) relative to male ones. Maximally uncoupled respiratory rate obtained upon adding CCCP was higher in mitochondria from female brain homogenate, providing further evidence for enhanced mitochondrial respiratory function in female brains.

Figure 2B,C, respectively, show the FCR and RCR parameters quantified for brain mitochondria in both sexes. Notably, when normalized by the ETS state (Fig. 2B), the consistently higher respiratory activity in female brain (Fig. 2A) disappeared. As shown by transmission electron microscopy analyses below, this may be due to increased mitochondrial biogenesis in female brains.

### Increased rates of H_2_O_2_ generation in mitochondria from male brain

Next, we explored sex differences in individual electron transport chain (ETC) complex contributions to ROS production by brain mitochondria. We show in Figure 2C,D that mitochondria from male brains generate significantly higher rates of H_2_O_2_ production during all NAD^+^‐linked resting and active respiratory states. This may be taken to imply that complex I is more tightly coupled in female brain mitochondria and is likely a source of ROS leakage in male brains. However, this male‐specific increase in ROS production during NAD^+^‐linked respiration was mostly abolished in the presence of succinate, suggesting that complex II is more ROS‐leaky in female mice brains. As also shown in Figure 2C, the rate of H_2_O_2_ generation during maximally uncoupled respiratory state was significantly higher in male compared to female brain mitochondria.

### Mitochondrial morphometric study in heart and brain of male and female mice

**Figure 3:**
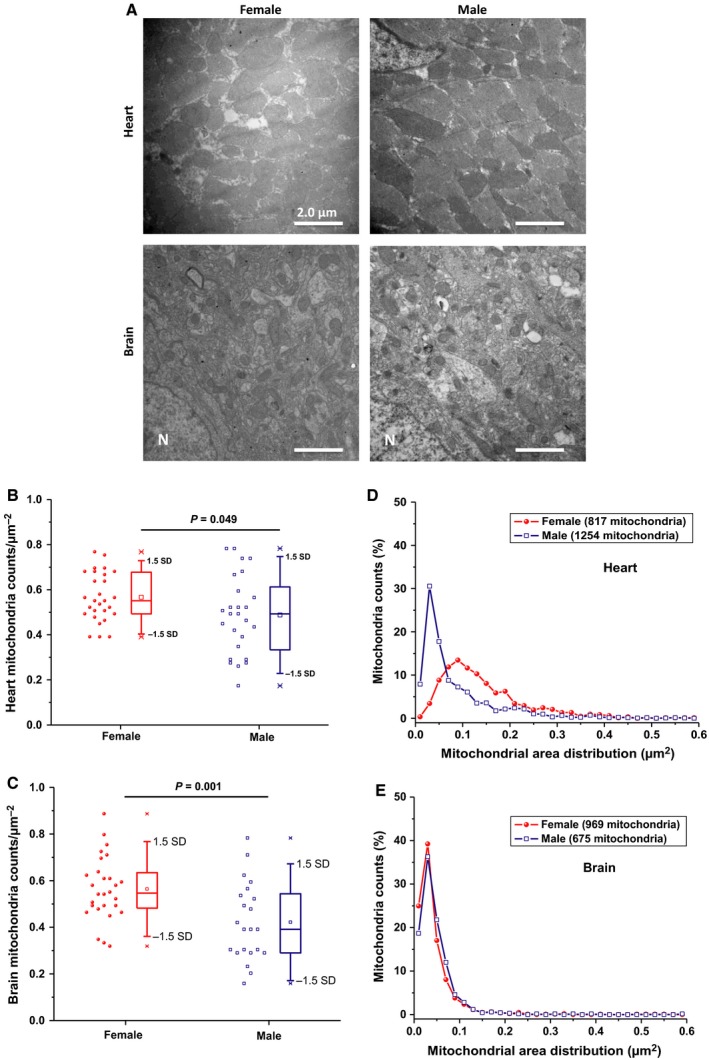
Sex‐related differences in mitochondrial counts and morphology in mouse heart and brain. (A) Mitochondrial density assessed by transmission electron microscopy in the left ventricular cardiac (upper two panels) or cortical brain tissue (lower two panels) from female (left) and male (right) mouse. Original magnification is ×10,000 and the white bars represent 2 μm scale. (B&C) Box and whisker + Data scatter plots depicting the quantification of cardiac and cortical mitochondria numbers per squared micron area of heart (B) or brain slices (C) from female versus male mice. Results represent the analysis of 5–7 images from four mice per group and One‐way ANOVA was carried out to determine statistical significance. Outliers are those data points lying outside ranges spanning 3 standard deviations. Analysis of heart (D) and brain (E) mitochondrial area distributions was carried out to infer mitochondrial fragmentation. Histograms of mitochondrial area versus counts were produced by splitting the area data range into bins of equal size (0.02 *μ*m^2^) for frequency counts. In (D&E), frequency counts were converted into percentage mitochondria population calculated per the total number of mitochondria per group and plotted against center bin values.

Due to dependence of mitochondria physiology on morphological features, the mitochondrial morphometric parameters were also studied in order to analyze sex‐related differences in mouse heart and brain, Figure 3. First, through direct counts per field, we found that female mice possess greater mitochondrial biogenesis in the heart (16.1 ± 4.5%, 28 slides from four females, 26 slides from four males, *P* = 0.049) and more in the brain (33.8 ± 3.6%, 28 slides from 4 females, 22 slides from four males, *P* = 0.001) as revealed by transmission electron microscopy (TEM), Figure 3A–C. Using TEM analyses, we also determined the area, perimeter, form factor, aspect ratio, and cristae density for each identified mitochondrion (see Table 1). Female heart showed a greater average size of mitochondria than males: significantly larger area (63%), longer perimeter (37%), slightly longer mitochondria (4%); a trend that was completely absent for brain mitochondria. The elongated phenotype in female's cardiac mitochondria indicates mitochondrial fusion, while the male's mitochondria were more rounded, indicating mitochondrial fission. Statistical histogram analysis of the area factor indicated that male's cardiac mitochondria are fragmented as revealed by greater number of categorical bins clustering toward smaller areas (Fig. 3D). Similar analyses on the form factor and aspect ratio implied that male's mitochondria are more round, not shown. This sex‐dependent mitochondrial fragmentation was absent in the brain (Fig. 3E).

**Table 1:** Effects of sex on mitochondrial morphometric parameters in mouse heart and brain

Morphological parameter	Mitochondria morphology parameters, mean ± SEM			
	Heart		Brain	
	Female	Male	Female	Male
Perimeter, *μ*m	0.731 ± 0.008	0.532 ± 0.007^a^	0.399 ± 0.006	0.417 ± 0.007
Circularity	0.795 ± 0.004	0.823 ± 0.003^a^	0.792 ± 0.004	0.799 ± 0.005
Form Factor	1.29 ± 0.01	1.24 ± 0.01^a^	1.32 ± 0.01	1.31 ± 0.01
Aspect Ratio	1.74 ± 0.02	1.60 ± 0.01^a^	1.84 ± 0.02	1.83 ± 0.03
Area, *μ*m^2^	0.145 ± 0.003	0.089 ± 0.003^a^	0.045 ± 0.001	0.047 ± 0.001

The data represent the mean ± S.E. and the analysis was recorded randomly from 676 male cardiac, 969 female cardiac, 1254 male cortical, and 817 female cortical mitochondria from four animals per group and were analyzed by One‐Way ANOVA test.

a Indicate *P *<* *0.01.

### No significant sex differences in NADPH‐Oxidase expression or activity in forebrain homogenate or in synaptosomes

**Figure 4:**
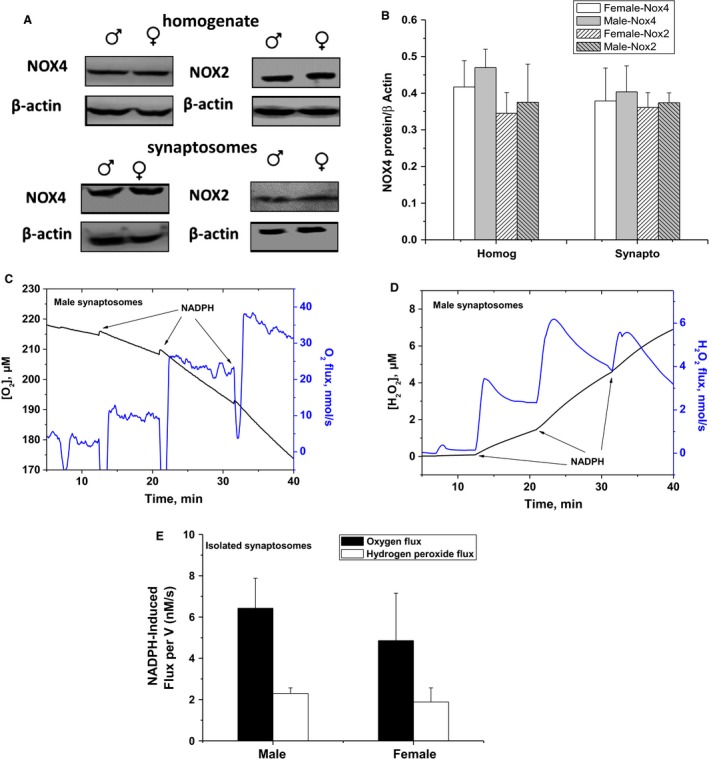
Lack of sex dependence of *Nox4 and Nox2 protein expression or activity in young mice forebrain homogenate and synaptosomes*. (A) Representative Western blot showing protein expression of the NADPH oxidase catalytic subunits of Nox4 and Nox2 enzymes in forebrain homogenate and synaptosomes isolated from male and female. (B) densitometric analyses of NOX proteins normalized by *β*‐actin in all cases. Values are calculated as relative intensity/*μ*g of loaded protein normalized by *β*‐actin expression and are given as mean ± SEM (*n* = 6–8). (C) NADPH oxidase activity is assessed by measuring oxygen consumption rate induced by adding NADPH substrate in its reduced form to a tightly closed Oroboros O2K chamber containing isolated synaptosomes. In parallel, NADPH‐induced NOX‐mediated H_2_O_2_ production was fluorimetrically monitored using HRP/Amplex Red system (D). (E) Three doses of NADPH were infused sequentially and the sum of the NADPH‐induced O_2_ flux was calculated after subtracting the routine oxygen flux. Means of 6‐7 animals/sex were calculated and presented as black bars and the sums of the maximum H_2_O_2_ flux reached during three successive additions of NADPH were averaged over *n* = 6–7 animals/sex and represented by the white bars.

In addition to mitochondria, NADPH oxidases are increasingly recognized as important players during development, in cerebrovascular diseases, in neurodegeneration, in psychological disorders, and in aging; recently reviewed in (Nayernia et al. 2014). In the central nervous system, Nox2 and Nox4 are reported as important enzymatic sources of ROS (Tang et al. 2012). To assess a possible sexual dimorphism in brain Nox‐mediated ROS generation at young age, we first analyzed Nox2 and Nox4 protein expression levels in both sexes. Western blot analysis revealed that Nox2 and Nox4 proteins in brain homogenate and purified synaptosomes were not sex‐dependent in young animals (*n* = 6–8 per group, Fig. 4A and B). We then explored if NOX activity in the brain is sex‐dependent using two independent assays. A useful neuronal model for investigating NOX activity is provided by isolated synaptosomes (Dugan et al. 2009). We have previously reported the detection of oxygen consumption by synaptosomal NOX using Oxygraphy (Behrens et al. 2007) and Oxygraphy in combination with EPR spin‐trapping spectroscopy (Dugan et al. 2009). Here, we utilized high‐resolution oxymetry (Oroboros O2k‐Station) to follow the effect of sex on NADPH‐induced oxygen consumption in isolated male and female synaptosomes. Additionally, we simultaneously used the O2k‐Fluoremeter, for the first time, to monitor the rate of hydrogen peroxide production in the same sample. This allowed us to distinguish between potential ROS sources based on designed combinations of selective substrates/inhibitors. In Figure 4, we show representative oxygen consumption (C) and hydrogen peroxide production (D) traces as induced by the addition of NADPH. No significant differences in oxygen consumption by synaptosomal NOX in male (*n* = 6) and female (*n* = 7) brains (Fig. 4E).

### A trend of elevated NOX‐dependent superoxide production in male brains

**Figure 5:**
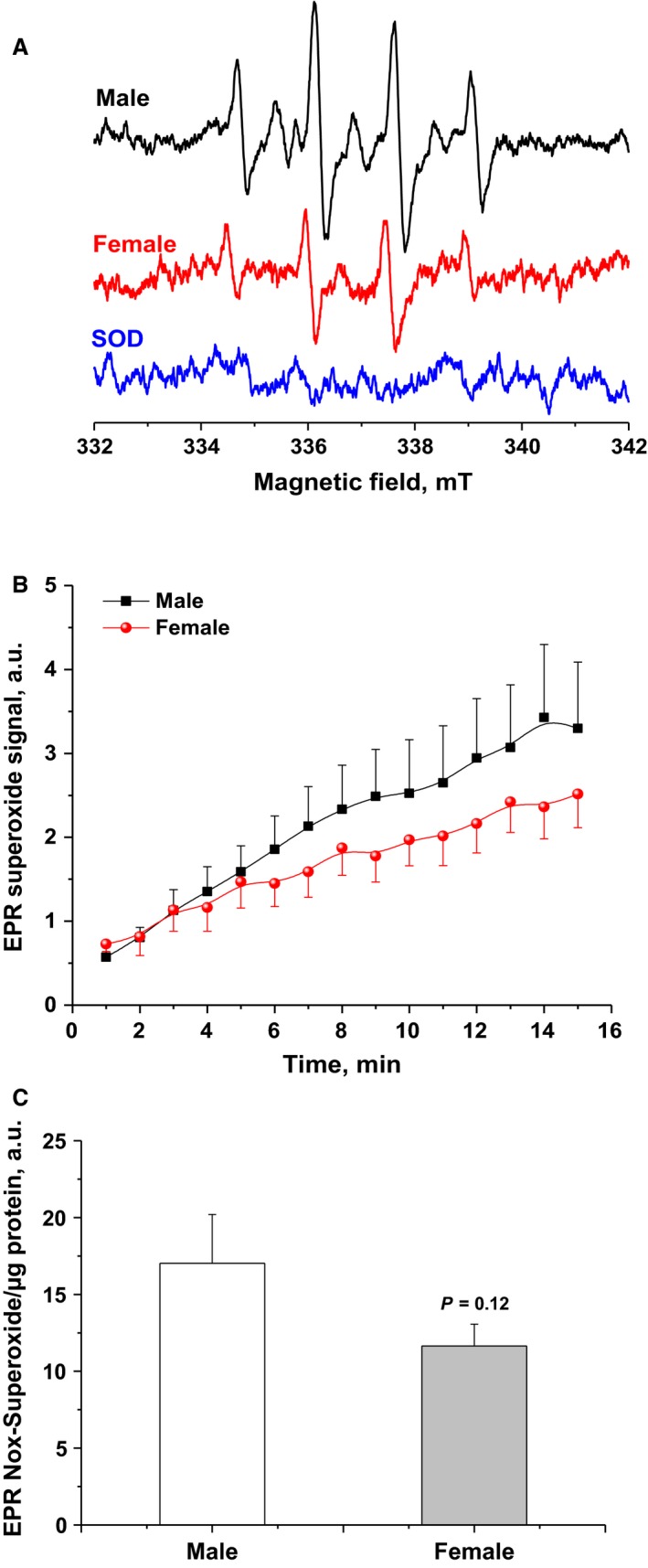
A trend of higher NOX‐mediated superoxide yield in isolated synaptosomes from male forebrains as assessed by EPR spin‐trapping spectroscopy. (A) Representative EPR spectra observed after 15 min from NADPH stimulation of NOX in female (red) or male (black) synaptosomes in the presence of DMPO spin trap. The observed signal is attributable to DMPO‐OH spin adduct that arises from superoxide‐DMPO as it was completely abolished by exogenous superoxide dismutase (blue spectrum). (B) Kinetics of NOX activity quantified by recording the amplitudes of the second low‐field peak after averaging two EPR spectra per minute in male (black) and female (red) synaptosomes. The shown kinetic traces are the average of 6‐8 animals/sex. (C) Quantifications of sex‐dependent superoxide yields by calculating areas under collected kinetic curves over 15 min of NADPH‐induced Nox activities. Values are given as mean ± SEM (*n* = 6‐8 animals/sex, *P* = 0.12).

To confirm the above results, we employed EPR spin‐trapping spectroscopy to measure NADPH‐induced activity in synaptosomes. The advantage of the EPR technique is its ability to detect, identify, and semiquantify various ROS simultaneously in real time (Dugan et al. 2009). Representative EPR traces are given in Figure 5A, which shows the activity of NOXs in synaptosomes through the detection of DMPO‐hydroxyl radical adduct that is originating from superoxide radical. The origin of the observed signal was confirmed by adding SOD, which completely eliminated the EPR signal of DMPO‐superoxide adduct (lower trace, Fig. 5A). Unexpectedly, a trend of higher superoxide production in male synaptosomal NOX was observed (8.49 ± 2.56 for males, *n* = 7; 4.19 ± 1.21 for females, *n* = 8; *P *=* *0.1, Fig. 5B and C). These results indicate that, although fluorescence‐detected H_2_O_2_ production was not different in both sexes, EPR detected relatively higher NOX‐induced superoxide production, which did not reach statistical significance in male brains (*P* = 0.1). We propose that this paradoxical discrepancy between superoxide and H_2_O_2_ trends may be due to sex differences in superoxide dismutase activity in synaptosomes; see below.

### Increased superoxide dismutase activity in female brain

**Figure 6:**
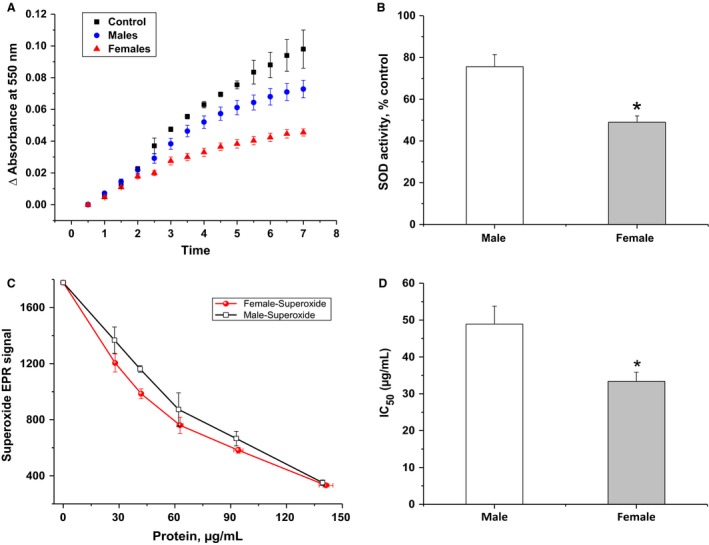
Superoxide dismutase activity in homogenized forebrain of female mouse is significantly higher than that in male brain. (A) Superoxide dismutase activity in male and female brains were determined by analyzing competitive superoxide reaction with ferricytochrome *c versus* SOD present in the studied samples. Spectrophotometric detection of SOD‐inhibitable reduction in cyt c by superoxide (generated by X/XO system) was followed at 550 nm in the absence (black trace) and presence of known protein concentrations of homogenized brain tissue samples from males (blue trace) or females (red trace). (B) Rates of superoxide‐mediated cyt c reduction in the presence of homogenates are shown as percentage of that in their absence. (C) An alternative assessment of SOD activity in male and female brains by EPR spin‐trapping spectroscopy. Controlled generation of O_2_^•^‾ by the X/XO system in the presence of DMPO spin trap allowed the quantification of superoxide yield by EPR in the absence and presence of different protein concentration of each. This resulted in dose–response curves, which were analyzed to obtain the IC_50_ values reflecting protein concentrations required to quench 50% of the controlled superoxide signals by each sample (represented in D). Values are given as mean ± SEM (*n* = 6 per group, *P <* *0.05).

To examine whether reduced NADPH‐dependent superoxide production in female synaptosomes described above, is due to differences between sexes with respect to antioxidant capacity, we assessed superoxide dismutase activity in male and female forebrains. SOD activity in brain homogenate was measured spectrophotometrically by monitoring the SOD‐inhibitable reduction of ferricytochrome *c* (cyt c) at 550 nm as described (Kuthan et al. 1982). Superoxide radical anion (O_2_^•^‾) was generated through enzymatic aerobic metabolism of hypoxanthine by xanthine oxidase. Fixed protein concentrations of homogenized brains from males or females were allowed to compete with cyt c for O_2_^•^‾ and the superoxide yields were calculated as areas under 7‐min acquisition curves (Fig. 6A). Relative to no‐tissue control, female homogenates reduced the superoxide accumulation significantly relative to the same amount of males' protein (75.59 ± 5.77% of control for males; 49.02 ± 3.00% of control for females; *n* = 6 per group, *P* < 0.01, Fig. 6B). This result indicates that SOD activity in female brains is higher than in male, which strongly supports a role of SOD activity in the apparent increase in ROS production in male brains.

To further confirm the role of superoxide dismutation capacity in female brains, we used a new approach to determine SOD activity in brain homogenate using EPR spin‐trapping spectroscopy. Since we are able to identify the involved radical(s) species through EPR analysis, this method can be used to determine SOD and/or peroxidase activities of a given sample. Here, we monitored the effect of increasing protein concentration of brain homogenates on the XO/X–generated superoxide EPR signal to evaluate the SOD capacity of the studied samples. Again, our results showed that female brain homogenate exhibits significantly higher SOD activity as compared to males (*P* < 0.05). The antioxidant activity was estimated by graphical analysis of the dose–response curves to calculate IC_50_ values which was 33.39 ± 2.47 *μ*g/mL for females and 48.92 ± 4.85 *μ*g/mL for males (*n* = 6 per group, Fig. 6C,D). Taken together, these results provide strong support to our hypothesis that the apparent higher ROS production in male's brain is due to its lower SOD capacity relative to that in female's brain.

## Discussion

While major progress has been made in understanding the influence of sex differences in free radical homeostasis in the context of aging (Ali et al. 2006; Sanz et al. 2007; Escames et al. 2013), sex differences in mitochondrial function and the dynamics of ROS at young ages are not extensively studied. Considering the reported involvement of mitochondrial dysfunction and oxidative stress in the pathogenesis of many disorders that begin early in life (Marazziti et al. 2012; Anitha et al. 2013), we carried out this study to explore if subtle sex differences in mitochondrial function and ROS homeostasis develop at young ages (2–4 months). Equipped with the highly stable Clark‐type oxygen electrode in the Oroboros O2k Station with its picomole sensitivity in addition to the fluorescence module designed to follow H_2_O_2_ in the same sample, we were able to interrogate each respiratory state while quantifying ROS leakage simultaneously. Importantly, these measurements were carried out in freshly prepared tissue homogenates; thus, insuring metabolically well‐preserved mitochondria that are closer to their physiology in vivo. The high integrity of the studied mitochondria is evident from high RCR values (>8) in both heart and brain, and was confirmed by the fact that exogenously added cyt c did not induce oxygen consumption indicating that the outer mitochondrial membrane was intact during sample homogenization.

In the heart, mitochondrial respiratory activity during resting (state 4) as well as phosphorylating (state 3) respirations did not significantly differ between young male and female mice. Sanz et al. (2007) reported similar lack of differences in respiratory activities between heart, skeletal muscle, and liver mitochondria isolated from middle‐aged male and female mice (10 months). Noteworthy, we found that normalized respiratory activity through complex II is generally higher than complex I, especially in males. Our data also showed that mitochondrial respiratory rate during maximum electron transport system capacity (maximally uncoupled state) in the heart is sex‐independent. Together, these findings suggest that, in the heart of young C57BL6 mice, mitochondrial respiratory function did not significantly differ between sexes. However, the rate of hydrogen peroxide generation from resting (*Leak‐I*) respiration via complex I and during ETS capacity was significantly higher in male's heart. It has been shown that dynamic change of mitochondrial morphology contributes to ROS overproduction and that mitochondrial fission/fusion machinery is implicated in acute and chronic production of ROS in the context of hyperglycemia (Yu et al. 2006). In support of this observation, we found by transmission electron microscopy analysis that male's cardiac mitochondria tend to exhibit fragmented phenotype.

Next, we investigated the mitochondrial respiratory function in male and female brains. In contrast to cardiac mitochondria, we found that mitochondrial respiratory activity is generally higher in female brains except during *Leak‐I* resting respiration via complex I. Our results are in tune with another study showing enhanced mitochondrial function in 5‐month‐old female SAMR1 mice (Escames et al. 2013). However, the observed greater respiratory rates in female brain were relinquished when internal normalization by ETS‐capacity was performed (Fig. 2C). This may be taken to reflect an increase in mitochondrial biogenesis in female brains. Sexual dimorphism in mitochondrial biogenesis has been previously reported (Sharma et al. 2014). Alternatively, estrogen involvement in enhancing mitochondrial respiratory function is widely reported. It has been demonstrated that mitochondria isolated from estrogen‐treated rats, exhibited significantly higher RCR due to enhanced oxygen consumption during state 3 respiration (Nilsen et al. 2006). The underlying mechanism of the modulatory effect conferred by estrogen on mitochondrial function was explained in terms of a pronounced upregulation in mitochondrial gene transcription (Murphy and Steenbergen 2007; Velarde 2014). Estrogen through its receptor, can upregulate the expression of nuclear respiratory factor (NRF1), which is a key transcription factor regulating transcription of majority of mitochondrial respiratory chain complexes. Moreover, estrogen can also modulate the transcription of NRF1 indirectly through interaction with another transcription factor known as peroxisome proliferator‐activated receptor gamma coactivator 1 (PGC‐1). Therefore, the enhanced mitochondrial respiratory function observed here may reflect an effect of estrogen on mitochondria biogenesis or on the transcription level of ETC proteins within female brain mitochondria. Using TEM analyses, we confirmed that, in average, female brains possess greater mitochondrial number relative to male's. Contrary to the heart, no sex‐dependent morphological differences have been registered in brain mitochondria. In effect, one may conclude that, in the heart, the quality of mitochondria matters, while in the brain, the number of mitochondria is more important to explain the observed respirator and ROS‐producing profiles.

In cells, mitochondria are usually proposed as important source of ROS production as well as being early victims of their own oxidative insults (Douglas et al. 2010)**.** Cellular respiration is a demand‐driven procedure, so that the rate of electron flux through the respiratory electron transport chain and hence oxygen consumption is governed by the rate at which protons leak through the inner membrane and reenter the mitochondrial matrix (resting respiration) or flow through ATP synthase to be used in ATP synthesis (active or phosphorylating respiration). It has been noted that ROS production during state 4 respiration is dramatically higher than during active respiration (Anderson et al. 2009; Ali et al. 2010). Attenuation of mitochondrial oxidative phosphorylation during state 4 will leave highly reduced ETC components, which is expected to leak more electrons to molecular oxygen. Indeed, our results indicated that state 4 respiration in male and female brains was consistently associated with higher ROS generation than state 3. Therefore, one would argue that slower respirations in male brains even during phosphorylating states may lead to the observed greater rates of ROS production in these animals. During state 4 respiration, addition of succinate causes electrons to flow from complex II to complex III. Under certain conditions, reverse electron transport, where electrons proceed backward from complex II to complex I, results in markedly higher rates of ROS production that are probably emitted at complex I (Schonfeld et al. 2010). Although the rate of ROS generation by complex I is significantly higher in mitochondria from male brains, this difference disappeared when the substrates for both complexes I and II were provided. Together, these results indicate that male brain mitochondria leak higher ROS than female, likely through complex I. The higher complex II respiratory activity in mitochondria from female brains reported above, suggest that succinate‐dependent H_2_O_2_ production is higher in female brain mitochondria, which might be due to back electron transfer from complex II to complex I (Schonfeld et al. 2010).

Generally, the higher rate of oxygen consumption observed in mitochondria from female brains, was associated with lower rates of ROS generation as compared to male brains. It is conceivable that enhanced antioxidant buffering capacity in female organs contributes to the observed lower ROS levels in comparison with organs from male. In this context, we tested the activity of SOD in male and female forebrains using two different approaches. Using cytochrome c assay, our results showed an enhanced activity of SOD enzyme in female brains relative to male brains. These findings were further confirmed by an assay developed in our laboratory for the determination of SOD activity using EPR spin‐trapping technique. Female brain homogenates displayed lower IC_50_ for superoxide elimination than male brain homogenates indicating that SOD activity in female is higher than that in male. Our results are consistent with earlier studies showing an enhanced SOD activity in female relative to male wistar rats hepatic mitochondria (Borras et al. 2003).

**Figure 7:**
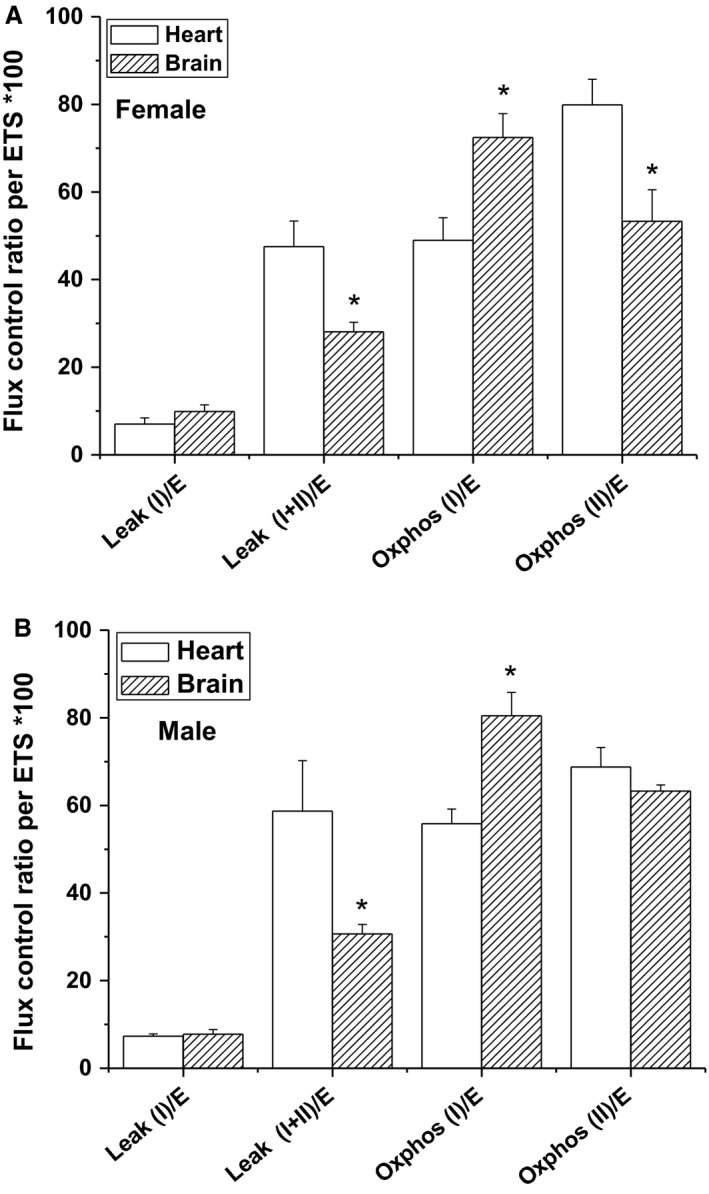
Organ‐dependent metabolic differences in males and females. Tissue‐specific flux control ratio (FCR) in female (A) and male (B) calculated as the ratio between individual respiratory states and Electron Transfer system (ETS) capacity of heart and brain mitochondria. Values are expressed as percentages (*n* = 6–8, *P < 0.05, brain vs. heart).

Interestingly, we have noted organ‐dependent changes in the FCR in both sexes; Figure 7. It is worth noting that, cardiac mitochondria from male and female mice displayed a significant increase in oxygen flux through complex I + II during state 4 respiration as compared to complex I (Leak (I)/E vs. Leak (I+II)/E), an effect that was not as pronounced in brain mitochondria (Fig. 7A and B). This implies that the activity of complex II (succinate dehydrogenase) in the heart is greater than in the brain. Similarly, FCR through complex II in female heart during state 3 respiration is significantly higher relative to complex I contribution (Oxphos (I)/E vs. Oxphos (II)/E). Both female and male brains showed the opposite trend where complex I contribution was significantly greater than complex II during active respiration. This is in agreement with a previous study showing that succinate dehydrogenase activity is higher in the heart than in the brain of female wistar albino rats (Mirandola et al. 2010). Therefore, our study provides a strong evidence for tissue‐specific respiratory differences eliciting complex II in the heart while eliciting complex I in the brain. Our findings are also in accordance with previous reports showing that mitochondrial respiration under physiological and pathological conditions are controlled by tissue‐specific mechanisms (Holmstrom et al. 2012).

It is increasingly recognized that mitochondria are not the only sources of ROS. NADPH oxidase has recently attracted a considerable attention as a key source of cellular ROS (Ali et al. 2010). We have previously reported that Nox2 is a major source of neuronal and synaptic superoxide generation in old brains (Dugan et al. 2009), and in ketamine‐induced psychosis (Behrens et al. 2007). Here, we followed the sex discrepancies in NOX activity and expression in brains of young male and female C57BL6 mice. Nox2 and Nox4 are two isoforms that are shown to be essential for ROS production in brain and many other organs. Quantification of Nox2 and Nox4 protein expressions in the homogenates of male and female brains revealed nonsignificant differences between sexes. Similarly, we found that Nox2 and Nox4 proteins are expressed at the same levels in synaptosomes isolated from male and female brains. We then assessed Nox activity using the Oroboros high‐resolution O2k Station. Following Nox activation by NADPH addition, similar oxygen consumption and hydrogen peroxide production rates by synaptosomal NOXs in both sexes were observed. This is the first report where NOX‐related rate of hydrogen peroxide production is monitored using the O2k. In parallel, NOX‐dependent superoxide production in synaptosomes was measured using spin‐trapping EPR spectroscopy. Contrary to our expectations, we found a strong trend of increased NOX‐derived superoxide in male synaptosomes, which may be attributed to the lower SOD activity in male brains. No previous study has investigated in detail whether sex affects NOX dynamics in young brains. Only one study assessed the expression of NOXs and its activity in the cerebral circulation (Miller et al. 2007). Therefore, and to the best of our knowledge, this is the first report that addresses in detail, the sex differences in neuronal and synaptic NOX expression and Nox‐dependent hydrogen peroxide as well as superoxide generation.

The underlying mechanism of the observed elevation of SOD activity in female brains may involve a neuroprotection conferred by the female sex hormone, estrogen. In fact, several studies demonstrated that sex differences in oxidative stress markers are estrogen dependent and that estrogen may exert protection in females by increasing antioxidant defenses and by diminishing ROS production (Vina et al. 2005). Interestingly, a cross talk between mitochondrial function and sex‐steroidal hormones during aging is reported (Velarde 2014). In addition, studies on systemic arteries of ovarectomized mice and in cultured cells, showed that exogenous estrogen downregulates some NOX subunits and inhibits NOX activity (Wagner et al. 2001; Florian et al. 2004). Furthermore, a study demonstrated that estrogen enhances the nuclear transcription of mitochondrial SOD2 by activating the MAPK and NF*κ*B pathway (Borras et al. 2005). Therefore, our data suggest that at young age, estrogen may be a fundamental factor that determines sex differences in mitochondrial function and morphology, ROS production, and antioxidant activity reported in this study.

Finally, our approach enabled us to revisit a fundamental question regarding the proportion of the mitochondrially utilized oxygen that converts into hydrogen peroxide during different physiologically relevant respiratory states. Mitochondrial superoxide formation occurs in the matrix (mainly from complex I) and on both sides of the inner mitochondrial membrane (from both complex I and complex III). While the O_2_^−•^ generated in the matrix from complex I is rapidly converted into H_2_O_2_, part of the O_2_^−•^ produced in the intermembrane space via the CoQ^•^ electron carrier may be trafficked to the cytoplasm via voltage‐dependent anion channels (Han et al. 2003). However, Mn‐SOD in the matrix and Cu,Zn‐SOD in the intermembrane space and in the cytosol directly convert O_2_^−•^ into H_2_O_2_. The uncharged H_2_O_2_ can move freely across mitochondrial and cytoplasmic membranes. We calibrated our H_2_O_2_ fluorescence signal using high‐resolution measurements of oxygen production due to selective decomposition of H_2_O_2_ by exogenous catalase. This allowed us to quantify the O_2_ proportions that are converted to H_2_O_2_ during each respiratory state in real time; see Table 1. It is generally discernable from the data in Table 1 that male mitochondria generate greater H_2_O_2_ proportions in both organs. Moreover, heart mitochondria in both sexes seem to be more tightly coupled with less ROS production except when complex I is involved in the actively phosphorylating states. Turrens predicted that the proportion of oxygen converted into O_2_^−•^ in vitro ([O_2_] ~ 200 *μ*mol/L) accounts for about 1‐2% of the overall oxygen consumption (Turrens 2003). However, our data indicate that during active *OXPHOS* states between 0.1 and 0.3% of the consumed oxygen are converted into H_2_O_2_ (Table 2). This percentage may exceed 2% only during resting respiration; that is, *Leak‐I* and *Leak‐II*. It remains to be tested if these proportions vary in aged mitochondria and under pathological conditions.

**Table 2:** Experimentally determined proportions of the utilized O_2_ that convert into H_2_O_2_ during all active, resting, and inhibited states of heart and brain mitochondria in both genders

State	H_2_0_2_, % consumed oxygen mean ± SEM			
	Female		Male	
	Brain	Heart	Brain	Heart
Leak‐I	1.63 ± 0.15	1.02 ± 0.13	2.70 ± 0.26	2.59 ± 0.36
Leak‐ll+R	1.36 ± 0.12	0.53 ± 0.05	1.86 ± 0.23	0.86 ± 0.12
OXPHOS‐I	0.12 ± 0.01	0.11 ± 0.01	0.25 ± 0.02	0.24 ± 0.03
OXPHOS‐II	0.28 ± 0.01	0.18 ± 0.02	0.48 ± 0.03	0.25 ± 0.03
AmA+R	7.89 ± 0.55	5.53 ± 0.70	6.05 ± 0.51	6.5 ± 0.60
ETS‐Capacity	0.08 ± 0.16	0.047 ± 0.006	0.16 ± 0.01	0.12 ± 0.02

ETS, Electron Transfer system.

In conclusion, we have been able to detect significant sex‐related differences in respiratory functions and morphology of heart and brain mitochondria in young animals. We simultaneously measured ROS production at each respiratory/inhibitory state. Additionally, we explored NOXs as potential ROS sources in the brain using multiple approaches. Finally, we assayed the antioxidant activity that is carried out by SOD which is a key enzyme that is ubiquitously distributed in different cellular compartments. Generally, female mitochondria were more effectively utilizing oxygen while producing smaller ROS amounts. NOX superoxide was also higher in male synaptosomes. Both female heart and brain exhibited significantly higher SOD activity. In light of published work, one can speculate that at young age, estrogen may be a fundamental factor that determines sex differences in mitochondrial function, ROS production, and antioxidant activity reported in this study. This study also provides an evidence that complex I is likely a source of ROS leakage in male brains while complex II is more ROS‐leaky in female mice hearts and brains. Abnormalities in the respiratory chain complex I and the resulting increased levels of ROS have been reported as crucial factors in the pathogenesis of several male biased neurological and cardiovascular disorders such as ASD (Giulivi et al. 2010), PD (Marella et al. 2009; Misiak et al. 2010), and ischemia reperfusion injury (Tompkins et al. 2006) and might represent metabolic features common to sexually dimorphic disorders of higher prevalence in males. In addition, there is a strong evidence that overproduction of ROS through mitochondrial respiratory complex II is critically involved in Rett syndrome pathogenesis (De Filippis et al. 2015), which is a female‐specific disease. Our results point in the same direction and may eventually assist in sex‐specific drug development aimed at improving mitochondrial functionality, and antioxidant capacity for treatment of sexually dimorphic disorders that occur early in life.

## Conflict of Interest

No competing interest declared for any of the contributing authors.
